# CRISPR-Cas systems target endogenous genes to impact bacterial physiology and alter mammalian immune responses

**DOI:** 10.1186/s43556-022-00084-1

**Published:** 2022-07-20

**Authors:** Qun Wu, Luqing Cui, Yingying Liu, Rongpeng Li, Menghong Dai, Zhenwei Xia, Min Wu

**Affiliations:** 1 Department of Pediatrics, Ruijin Hospital affiliated to Shanghai Jiao Tong University School of Medicine, Shanghai, 200025 China; 2 Department of Biomedical Sciences, School of Medicine and Health Sciences University of North Dakota, Grand Forks, North Dakota 58203-9037 USA; 3 The Cooperative Innovation Center for Sustainable Pig Production, Huazhong Agricultural University, Wuhan, Hubei 430070 P. R. China; 4 Key Laboratory of Biotechnology for Medicinal Plants of Jiangsu Province, School of Life Sciences, Jiangsu Normal University, Xuzhou, 221116 China

**Keywords:** Inflammasome, Host immune response, Endogenous gene targeting, Phagocytosis, Biofilms, Inflammatory response

## Abstract

CRISPR-Cas systems are an immune defense mechanism that is widespread in archaea and bacteria against invasive phages or foreign genetic elements. In the last decade, CRISPR-Cas systems have been a leading gene-editing tool for agriculture (plant engineering), biotechnology, and human health (e.g., diagnosis and treatment of cancers and genetic diseases), benefitted from unprecedented discoveries of basic bacterial research. However, the functional complexity of CRISPR systems is far beyond the original scope of immune defense. CRISPR-Cas systems are implicated in influencing the expression of physiology and virulence genes and subsequently altering the formation of bacterial biofilm, drug resistance, invasive potency as well as bacterial own physiological characteristics. Moreover, increasing evidence supports that bacterial CRISPR-Cas systems might intriguingly influence mammalian immune responses through targeting endogenous genes, especially those relating to virulence; however, unfortunately, their underlying mechanisms are largely unclear. Nevertheless, the interaction between bacterial CRISPR-Cas systems and eukaryotic cells is complex with numerous mysteries that necessitate further investigation efforts. Here, we summarize the non-canonical functions of CRISPR-Cas that potentially impact bacterial physiology, pathogenicity, antimicrobial resistance, and thereby altering the courses of mammalian immune responses.

## Introduction

**Fig. 1:**
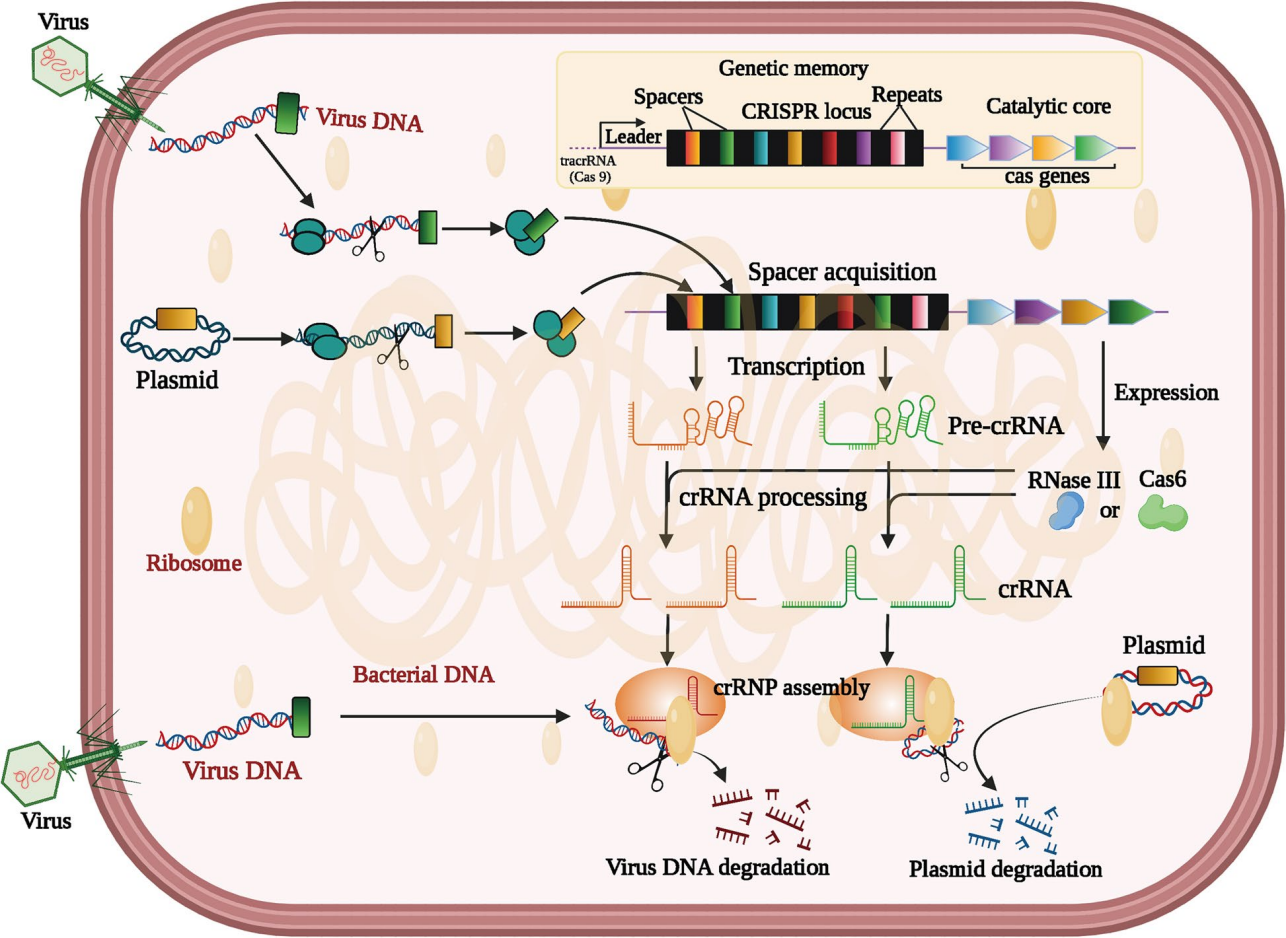
Overview of the CRISPR-Cas systems. CRISPR-Cas systems including leader, spacer and repeats are alternately arranged to form R-S structure and *cas* locus. The function of CRISPR-Cas systems include three stages: spacer acquisition, crRNA processing and assembly, and target degradation. The first step occurs after the foreign DNA sequence invades bacteria, and the bacterial genome intercepts a sequence from the invaded DNA fragment and integrates it into its CRISPR sequence to become a new spacer. Then, CRISPR clusters are transcribed into pre-crRNA under the initiation of the leader and further processed into mature small crRNAs. The mature crRNAs and Cas proteins assemble to form a CRISPR ribonucleoprotein (crRNP) complex. Once the same foreign DNA invades, the Cas protein complex binds, cleaves and degrades it by the base-pair principle

Clustered regularly interspaced short palindromic repeats (CRISPR) and their associated (Cas) proteins possess a unique structural and functional entity in bacterial genomes [1]. Thus far, CRISPR-Cas systems are the sole recognized acquired immunity against invading genetic elements found in many bacterial species and in most archaea. According to the CRISPR database (http://crispr.i2bc.paris-saclay.fr), CRISPR arrays are found in 202 (87%) out of 232 analyzed archaeal species and 3059 (45%) out of 6782 bacterial species by genome sequencing studies [2, 3]. The CRISPR-Cas systems consist of four parts: CRISPR regions, leader sequences, Cas proteins and tracrRNA (for Cas9) [4, 5]. The action steps of CRISPR-Cas systems are generally described as following three stages: adaptation, crRNA expression and interference (Fig. 1). The *cas* genes locating near the CRISPR locus encode CRISPR-related proteins to exert adaptive immune defense against bacteriophages or other genetic elements [6].

According to the diversity of *cas* genes and the mechanism of action for Cas proteins, CRISPR-Cas systems are categorized into 2 classes and further classified into six different types: type I to type VI [7, 8]. Basic CRISPR research only constitutes a relatively small portion, and the majority of work rather focuses on the application of CRISPR-Cas systems in genome-editing, transcriptional control, biotechnology, and agriculture engineering [9–15]. Furthermore, how CRISPR-Cas systems interact with other genes in the prokaryote, such as endogenous gene targeting and virulence gene regulation, remains largely un-elucidated [16–20].

**Fig. 2:**
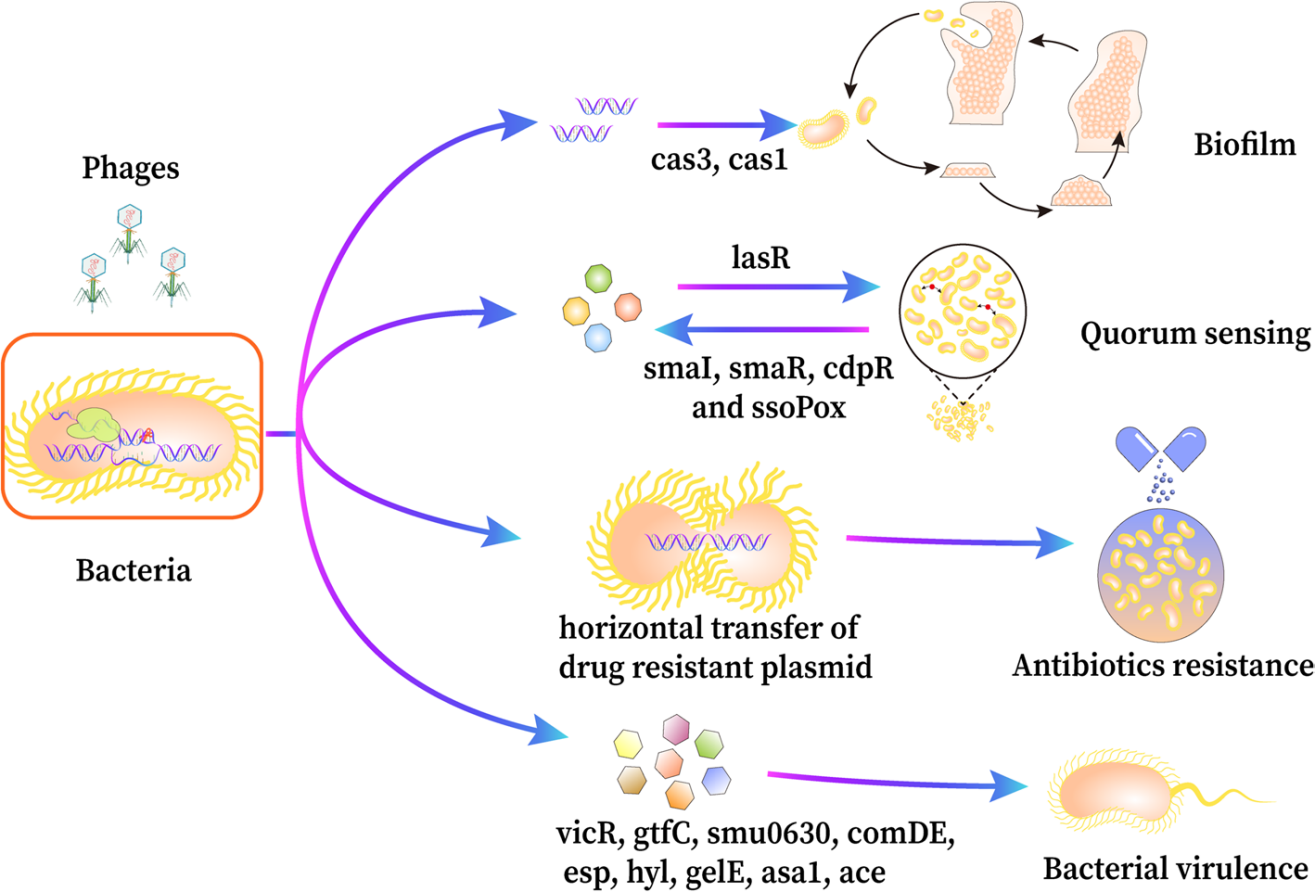
Effect of CRISPR-Cas systems on physiological traits of bacteria. CRISPR-Cas systems affecting multiple bacterial characteristics, such as virulence, bacterial biofilm, QS and antibiotics resistance. CRISPR-Cas systems are associated with the expression of multiple virulence factors, such as *vicR, gtfC, smu0630, comDE* (*Streptococcus sanguinis*), *esp, hyl, gelE, asa1, ace*(*E. faecalis*). cas3 and cas1 genes directly or indirectly participate in and affect the formation of bacterial biofilm. There is mutual regulation between CRISPR-Cas systems and QS systems, and many genes are involved in this process, such as *LasR, SmaI, SmaR cdpR, SsoPox* and *SmaR*. CRISPR-Cas systems regulate the transfer of drug-resistant plasmids

Apart from providing immunity, CRISPR-Cas systems have been shown to influence the expression of bacterial genes that potentially impact bacterial biofilm formation, quorum sensing, antibiotic resistance, virulence (Fig. 2), adaptability, and viability of bacteria to environmental changes [21–25]. In addition to influencing bacterial behaviors, CRISPR-Cas systems might also have a role in influencing the immune systems of eukaryotic cells, helping bacteria evade host defense mechanisms. In this article, we will discuss some latest advances in the effects of CRISPR-Cas systems on the physiological and virulence alteration of bacteria and potential mechanisms as well as the impact of CRISPR-Cas-mediated gene regulation on mammalian host response, more detailed coverage of other relevant CRISPR-Cas researches may be found elsewhere [26–28].

## CRISPR-Cas systems regulate bacterial biofilm formation

### Biofilms and their function

Biofilms are structural surface materials of microbial cells encased in an extracellular matrix that may substantially decrease the susceptibility to antimicrobial agents, hence the potency for forming biofilms is critical for virulence and drug resistance [29]. Bacteria secrete a large amount of extracellular polymeric substances (EPS) to form biofilm, which can hinder the penetration of antibiotics and produce drug resistance. In addition, the virulence of bacteria in high density increases sharply under the regulation of the sensing system disrupts host immune systems, and gains tolerance to antibiotic therapy, ultimately leading to a variety of severe or persistent infections at the mammalian mucosal surface (e.g., respiratory or gut systems of mice and humans) [30, 31].

### CRISPR-Cas systems alter biofilm levels

Recently, growing studies have shown that CRISPR-Cas systems can also regulate the formation of biofilm. CRISPR systems affect the formation of bacterial biofilm, thus affecting the transmission, toxicity and control of pathogenic bacteria. For example, *Enterococcus faecalis* carrying CRISPR-Cas systems possesses more prominent biofilm forming ability, better resists the destruction of bacteria by external factors, and effectively improves the colonization efficiency of bacteria in the host [32]. The resistance of *E. coli* to phages in Stx2 phage lysogen is significantly enhanced at a high bacterial density, while the biofilm-forming ability of *E. coli* is markedly decreased due to the activated CRISPR-Cas system mediated by mutant H-NS (heat-stable nucleoid structuring) protein which exist in a variety of Gram-negative bacterial species and inhibit gene transcription [33].

Another study showed that *E. faecalis* SK460 strains with strong biofilm forming ability in *E. faecalis* lack a Cas gene and functional CRISPR system. A possible reason is that the absence of CRISPR-Cas systems promotes the acquisition of antibiotic resistance gene, pheromone response plasmid, prophage, and pathogenic island.

Likewise, clinical studies suggest the same results: a study showed that the *E. faecalis* root canal isolates without CRISPR-Cas systems exhibited stronger biofilm formation and significant periapical lesions and strains lacking CRISPR1 or CRISPR3 loci produced increased biofilms compared to the strains containing CRISPR1 or CRISPR3 loci [25, 34]. This study suggests that there is an interaction between CRISPR-Cas systems and genes that govern bacterial biofilm formation.

### Mechanisms for CRISPR-Cas-mediated biofilm regulation

After phage DMS3 infection, *Pseudomonas aeruginosa* as one of the lysogenic bacteria could not form biofilm properly. A further study demonstrated that when the bacteria have a complete CRISPR system and the spacer sequence of CRISPR structure cannot effectively combat DMS3-42, a phage that can inhibit the biofilm formation in lysogenesis condition. These results suggest that CRISPR-Cas systems may regulate gene expression through partial base-pairing mechanisms during biofilm formation [35]. This base-pair principle is the key to initiating the activation of CRISPR-Cas activity that may either result in anti-phage immunity or self-targeting that may regulate internal gene expression but potentially cause side effects [36–38].

Importantly, the existence of the protospacer-adjacent motif (PAM) in the *P. aeruginosa* genome is essential for the CRISPR-dependent loss of biofilm features, suggesting that endogenous targeting may share the same mechanism as anti-phage immunity defense. Wild-type (WT) *P. aeruginosa* PA14 lost the ability of forming biofilm after infected by lysogenic phage DSM3. Interfering with CRISPR2 (CRISPR array 2) or eliminating five of the six *cas* genes in *P. aeruginosa* PA14 Type I-F can restore the formation of biofilm. The study strongly suggests that the loss of biofilm is closely related to CRISPR and *cas* genes [39]. Also, a CRISPR-specific insertion sequence is needed to inhibit biofilm formation of *P. aeruginosa* as transcription of antisense RNA and silencing of the relevant genes is implicated in altered biofilm formation [39, 40]. Experiments showed that CRISPR-Cas systems in the bacterium were required for inhibiting the formation of biofilm and the ability of bacterial aggregation [39]. In addition, the study found that CRISPR2 instead of CRISPR1 plays a pivotal role in restoring biofilm formation to DMS3 lysogens [40]. These studies support the view that CRISPR-Cas may function in either inhibiting or promoting the formation of biofilms in different bacteria and depending on the conditions. Nevertheless, the detailed mechanisms of how CRISPR-Cas systems affect biofilm formation is currently unclear and needs to be further explored.

To further test this hypothesis and define the mechanisms of CRISPR-Cas in regulating biofilms, the impact of CRISPR-Cas is evaluated at the single Cas level. For instance, a *cas3* gene deletion mutant of *Streptococcus mutans* UA159 showed impaired biofilm formation and weakened competition against (co-cultured) *Streptococcus sanguinis* in the presence of fluoride by up-regulating virulence genes (*vicR*, *gtfC*, *smu0630* and *comDE*) [41]. This study illustrates that *cas3* of *S. mutans* may be a positive modulator of biofilm formation and fluoride resistance. Other *cas* genes, such as *cas1* encoded integrase, participate in the acquisition of a new DNA sequence from invading phages and indirectly affecting the formation and aggregation of bacterial biofilm [39]. Alternatively, self-targeting CRISPR arrays in the genome of *Streptococcus mutans* (a common oral pathogenic bacterium) that can target bacterial virulence genes gtfB or gtfBgtfC may lead to the decrease of EPS and disrupt the biofilm formation [42].

### Summary for CRISPR-Cas in biofilm formation

The above studies together indicate that different components of CRISPR-Cas systems could impact the formation of bacterial biofilms by influencing the expression of distinct genes. CRISPR-Cas-mediated regulation of biofilm formation, aggregation, and motility may be critical mechanisms to limit the transmission of phages among bacteria. In other words, phage-infected bacteria may help separate themselves from biofilm and other colonization sites, which may play an important role in preventing large-scale bacterial infection, a potential self-limiting mechanism to help maintain their stable equilibrium [43, 44].

## CRISPR-Cas systems interact with quorum sensing systems

### Quorum sensing (QS) systems

QS systems are a powerful communication mechanism for adjusting bacterial population density, which relies on signal molecules, such as autoinducers (AI). QS systems are potential gene regulators that powerfully control hundreds of genes to directly or indirectly impact various aspects of bacterial physiology features and virulence potency, including cell adherence, motility, and biofilm formation, which are important factors for bacterial growth and migration in normal or adverse environments [45–47]. Sophisticated transcriptome and proteomics analyses suggest that QS systems are not only a local regulatory mechanism but also controlling global bacterial behaviors, including aforementioned virulence characteristics [48]. Similarly, QS also plays an important regulatory role in Gram-negative bacteria, controlling the collective behavior of Gram-negative bacteria by secreting signal molecules [49]. Increasing studies have shown that bacterial biological characteristics are strongly associated with the QS systems; for example, biofilm-forming bacteria use QS to up- or down-regulate gene expression that may be related to CRISPR-Cas systems and will be discussed in more detail in next sections [50, 51] .

### CRISPR-Cas systems may sense QS systems

Shortly after discovering Cas9 gene-editing potential, CRISPR-Cas systems (Type I-F) of *P. aeruginosa* were implicated in impacting various bacterial functions [52, 53]. As biofilm formation is primarily regulated by QS systems, roles of CRISPR-Cas in biofilm formation may be related to QS as reported in QS in *Serratia* sp. ATCC39006 strain [54, 55], which may be independent of *N*-acyl homoserine lactone (AHL). In contrast, some homologous proteins of AI receptors LuxIR, such as SmaI and SmaR, influence both the adaption and interference process of CRISPR-Cas as well as expression of *cas* operons in *Serratia* sp. [54]. Through the electrically-controlled CRISPR-based transcriptional regulation, the activating effect of CRISPR-Cas by QS is also observed in another bacterium, *E. coli*, facilitating communication between bacterial cells [56]. In Gram-negative bacteria, such as *P. aeruginosa* and marine bacterium *Chromobacterium violaceum*, QS interfering enzymes lactonase SsoPox may down-regulate the expression of CRISPR-Cas, providing indirect evidence for QS reciprocal effects on CRISPR-Cas [57]. Recently, RNA-seq analysis found that the expression of Type I-F CRISPR-Cas was significantly increased in QS mutant strains ΔlitR of *Aliivibrio wodanis*, implying that LitR, a master QS regulator, negatively affected Type I-F CRISPR [58]. These findings suggest that *cas* genes are regulated by QS systems when bacteria reach to a high density, then leading to increased integration of spacer sequences of exogenous DNA fragments, and thus heightening the CRISPR-Cas-mediated resistance to invasion of phages and other genetic elements.

### Interaction between QS and CRISPR-Cas

Given the above-mentioned discussion, there is likely mutual adjustment between CRISPR-Cas and QS systems. The adaptive immune function of CRISPR-Cas is often inhibited when the QS signaling pathway is absent or blocked, suggesting that QS systems play an indispensable role in CRISPR-Cas activation [59]. Consistent with this conception, *cdpR*, a QS negative regulatory gene, impeded the transcription of *cas* operon via interrupting the binding of virulence factor regulator to *cas1* promoter induced by LasI/RhlI-mediated autoinducers, dampening Cas3 nuclease activities [60]. Mechanistically, small regulatory RNAs in bacteria may augment CRISPR levels to facilitate the function against phage infection, which is targeted on a leader sequence to negatively affecting Rho-mediated transcription termination [61]. Collectively, these findings indicate that QS-mediated CRISPR-Cas regulation may be a common phenomenon within the bacterial signaling network but much of mechanistic detail remains undetermined and requires further study. The QS regulatory pathway serves as homeostasis mechanism to maintain the balance between phage infection risk and CRISPR-Cas quantities, and contributes to the precise control of the adaptive immunity in bacteria [54, 59].

Based on above-discussed observations, we speculate that QS systems and CRISPR-Cas systems may interact each other to regulate the expression of various genes and alter bacterial behaviors, including immunity and virulence. For example, QS may effectively modulate the expression of CRISPR-Cas systems and thereby altering their function. In return, CRISPR-Cas systems render strong feedback to QS systems by influencing the expression and function of some critical QS regulators, such as the *lasR* gene, which may have far-reaching effects on the physiology and virulence potency of the involved bacterium [24]. Additionally, we have found that deletion of *cas3* in *Salmonella enterica* induced significant upregulation of QS systems by using the transcriptome-wide screen (RNA-Seq) [62].

While the interaction between CRISPR-Cas and QS systems may be well documented, the regulatory pathways and molecular mechanisms of QS and CRISPR-Cas activation remain to be fully elucidated [63]. Further exploring the mutual regulation mechanism in broader scopes and deeper levels (e.g., diverse species and strains as well as many different signaling pathways) will be necessary to improve our knowledge of how CRISPR-Cas and QS systems are intertwined to control bacterial physiology and virulence.

## CRISPR-Cas systems modulate bacterial pathogenicity

### CRISPR-Cas systems potentially adjust bacterial pathogenicity

Next, we will discuss how some of the above-mentioned regulations impact bacterial pathogenicity (Table 1). A series of studies indicate a potential link between CRISPR-Cas and bacterial virulence, which may directly or indirectly alter the course or extent of host defense in mammals. A mouse urinary tract infection model with *Enterococcus faecalis* isolates [32, 73] demonstrated that type II CRISPR-harboring strain manifests increased mortality compared to the CRISPR negative strain as a control. In addition to influencing the genomic differences that lead to bacterial pathogenicity through two non-mutually exclusive processes, CRISPR-Cas also blocked the recognition of exogenous DNA mobile elements carrying virulence factors (endotoxin, exotoxin, or drug resistance genes), thereby reducing bacterial virulence and limiting potential secondary infections [32]. Compared to the attenuated strain of *C. jejuni*, highly virulent strains bear a shorter or complete absence of CRISPR sequence, indicating that deleting CRISPR increases virulence and leads to severe enteritis and serious complications of infection [70].

**Table 1:** Some examples of endogenous targeting by CRISPR-Cas system

Species	CRISPR-Cas type	Function	Mechanism	Experimental evidence	References
*Enterococcus faecalis*	Type II-A	Bacterial mortality	Blocking the recognition and acquisition of exogenous DNA mobile elements carrying virulence factors.	No	[32, 64, 65]
*Francisella novicida*	Type II-B	Innate immune evasion Envelope permeability	Repressing *blp* mRNA levels, Cas9 mediates evasion of TLR2, promoting bacterial virulence; Increasing inflammasome activation.	Yes	[21, 26, 66]
*Neisseria meningitidis*	Type II-C	Adhere to, invade, replicate, and survive in human epithelial cells	Indirect regulation	No	[21, 67]
*Pseudomonas aeruginosa*	Type I-F	Innate immune evasion Pro-inflammatory responses of the host in cells and mice	Targeting mRNA of the bacterial quorum-sensing regulator LasR to dampen the recognition by TLR4; Inducing inflammasome activation via regulation of autophagy.	Yes	[24, 68]
*Campylobacter jejuni*	Type II-C	Biofilm formation Invasion and intracellular survivability Swarming	Indirect regulation	No	[69, 70]
*Salmonella enterica*	Type I-E	Biofilm formation Cell infection Animal pathogenicity	Impacting a series of genes related to QS, the flagellum, and the SPI-1-T3SS system.	No	[62]
*Legionella pneumophila*	Type II-B	Intracellular growth Intracellular infection of amoebae	RNase activity of Cas2 related to virulence mRNA expression by activated RNase.	No	[71, 72]
*Streptococcus mutans*	Type I-C	Biofilm formation Fluoride sensitivity	Regulating genes that are related to biofilm formation.	No	[41, 73]

CRISPR-Cas systems might either enhance or inhibit the virulence of divergent bacteria depending on different situations. Patients who were infected with *Klebsiella pneumoniae* bearing the type I-E^*^ CRISPR-Cas (both Cas1 type B and Cas3 type B alleles positive) system showed higher intensive care unit (ICU) occupancy, mortality rates and higher virulence gene clustered in comparison with patients infected with CRISPR-negative isolates [64]. Deletion of *cas3* gene (the representative nuclease of the class 1 type I CRISPR-Cas system) in *Porphyromonas gingivalis*, increased invasive potency in human monocyte/macrophage cell line THP-1 [74]. Furthermore, research illustrated that CRISPR-Cas could prevent the acquisition of certain pathogenic factors in some *E. faecalis* isolates to influence its pathogenic traits [65]. Collectively, these findings argue that CRISPR-Cas systems indeed shape the host responses through a spectrum of distinct mechanisms in experimental conditions and/or potentially clinical scenarios.

### CRISPR-Cas systems are likely associated with virulence regulation

Koonin and colleagues unveiled that prokaryotic toxins are closely associated with CRISPR-Cas, which induce either bacterial death or dormancy in the case of an immune system failure [75]. During the study of 81 strains of Shiga toxin producing *Escherichia coli* (STEC), Magaly et al. found that the amount of bacterial spacer sequences was inversely correlated with the severity of the disease, the fewer the spacer sequences, the higher the pathogenicity. The reason is that, the less CRISPR-*cas* system activity, the lower the chance to acquire pathogenicity features. In addition, the author found no relevance between the presence of subtype I-E *cas* and virulence genes, suggesting that these events may prevent the uptake and acquisition of virulence genes. Therefore, CRISPR array length and virulence genes are likely to be associated with other factors, which need to be further evaluated [76]. *Vibrio cholerae* in the GI tract could encode CRISPR-Cas genes along with the T6SS (VgrG, Hcp, and PAAR), indicating the mobility of CRISPR-Cas and its relevance to bacterial virulence [77].

However, another study reached conflict conclusions: genotypical screening based on PCR approaches investigated relationship between CRISPR elements and virulence genes in *E. faecalis*, including *ace*, *asa1, cylA*, *efaA*, *ebpR*, *esp*, *gelE*, and *hyl*. The researchers found that the presence of CRISPR loci seemed not relevant to the decrease of the number of pathogenic factors and found that CRISPR1-*cas* deficiency was only related to *esp, cylA, asa1* gene deficiency. These discoveries indicate that CRISPR loci may partially repress the expression or function of virulence factors and Pathogenicity Island [65].

## CRISPR-Cas systems impact endogenous genes (bacterial own genome) to Alter host immune responses

### Endogenous targeting by type II CRISPR-Cas

**Fig. 3:**
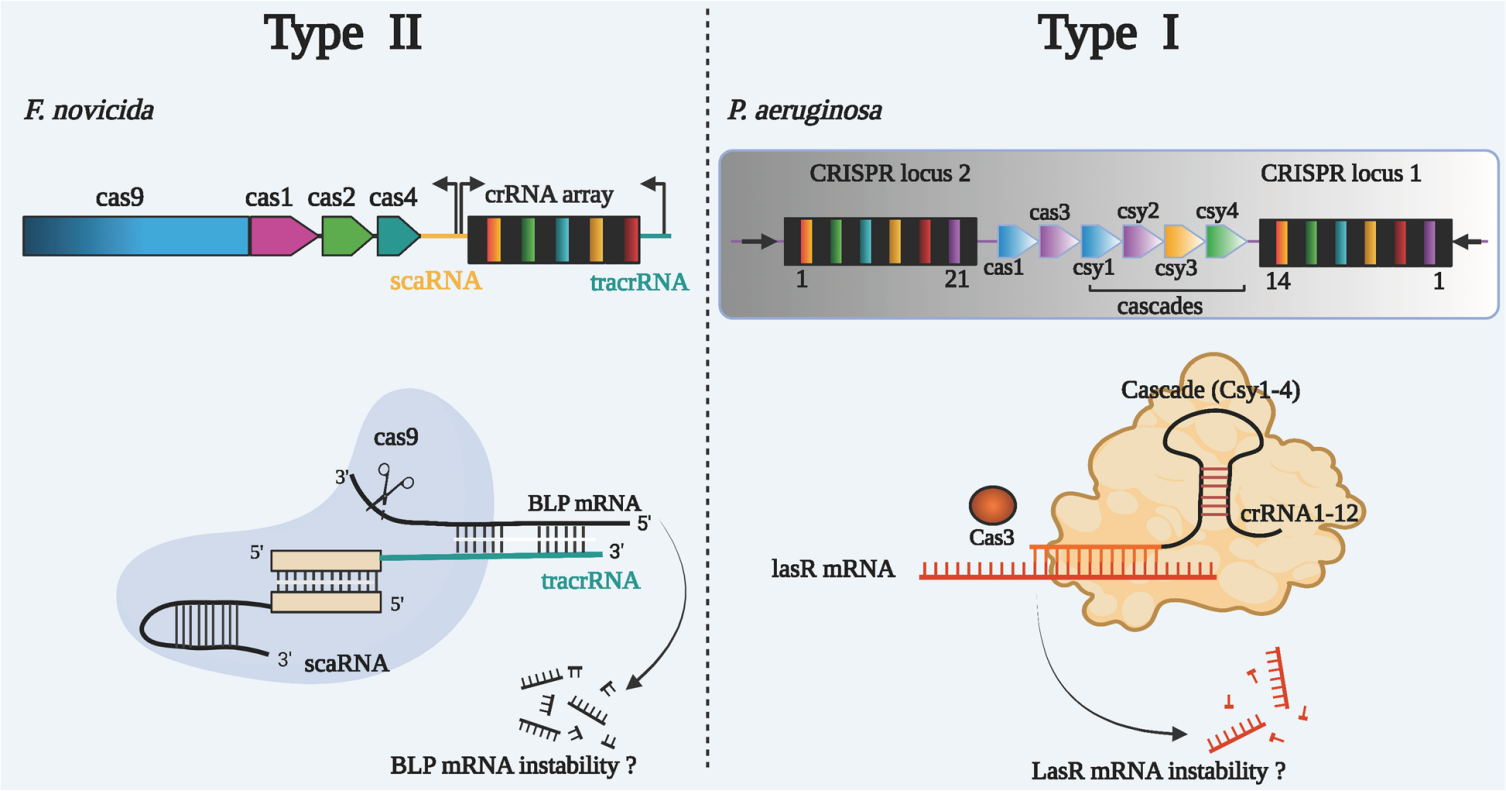
Type II and type I CRISPR-Cas systems adjust the expression of endogenous transcripts. TracrRNA and scaRNA (small, CRISPR-Cas-associated RNA) form a dual-RNA complex through sequence recognition to the CRISPR repeats. This dsRNA structure enables the free terminal of the tracrRNA to interact through a non-identity matching mRNA encoding the BLP, the stability of BLP mRNA is impeded and thereby leading to the degradation of mRNA. Type I CRISPR-Cas system of PA14 targets the mRNA of the QS regulator LasR. The crRNA1-12 structure is associated with Cascade (Csy1-4 complex) and interacts with *lasR* mRNA through a sequence complementing a part of crRNA1-12, and resulting in lowered *lasR* mRNA stability

In addition to targeting foreign mobile genetic elements, CRISPR spacers also target self-sequences. This self-targeting (autoimmunity) may be harmful to the stability of bacterial host genome, but it is not fatal under specific circumstances [78]. Increasing evidence suggests that Cas9, abundant in the genome of pathogenic bacteria, regulates virulence factors in several bacteria [21]. The Type II CRISPR-Cas systems of *Francisella novicida* are implicated in influencing bacterial growth, differentiation and virulence, and thereby leading to altered host defense by decreasing the expression of bacterial lipoprotein (BLP), a pathogen-associated molecular pattern (PAMP) antigen (Fig. 3) [21].

This study as a pilot test indicates that CRISPR-Cas systems contribute to virulence regulation in bacteria and hence influence their survival in the natural hosts [66]. Furthermore, Cas9 may influence the adherence of bacteria to the surface of host cells to modulate the composition and structure of bacterial envelope [21]. Recently, another study uncovered the loss of adherence to human nasopharyngeal epithelia by a *cas9* deletion meningococcus mutant may be dependent on the indirect regulation instead of influencing adherence genes’ expression [67]. Hence, the meningococcal CRISPR-Cas systems may exhibit novel functions beyond their classical duty in bacterial host defense against foreign DNA [67].

### Endogenous targeting by type I CRISPR-Cas

Type I CRISPR-Cas systems of *Pseudomonas* PA14 were shown to alter the levels of *lasR* mRNA to influence the immune response of macrophages. This work found that the *lasR* mRNA region (648 to 687) contains critical homologous sequences to part of spacers (Type I CRISPR array), and that the first 2 nt of the 5′-GGA-3′ site serves as key PAM-like nucleotides for this targeting. As Type I CRISPR-Cas decreased *lasR* mRNA stability, bacteria evaded the recognition and clearance of TLR4-mediated response by host immune cells, thus resulting in lowered inflammatory response [24] (Fig. 3).

Hence, the type I CRISPR-Cas systems may indirectly influence host defense by influnencing inflammasome activation via regulation of autophagy or other unknown signaling pathways [68]. According to the phenotypic determination of virulence characteristics and transcriptome analysis, it can be speculated that CRISPR-*cas9* is involved in regulating a large number of virulence related genes, such as downregulating membrane coding genes, lipoprotein genes and flagellum related genes. Compared to WT bacteria, the Δ*cas9* mutant showed a decrease in four aspects: mobility, biofilm formation ability, intracellular viability and toxin production ability. Thus, Cas9 contributes to the virulence of *C. jejuni* [69]. Interestingly, the expression of Cas9 protein in strains lacking CRISPR loci also increased the virulence of bacteria, suggesting that the function of Cas proteins may be exerted through several distinct mechanisms, some of them likely independent of CRISPR transcript levels [70]. It has been reported that cas9 cannot only target DNA, but also cleave endogenous RNAs to influence gene inactivation or activation [79].

### CRISPR-Cas endogenous targeting and autoimmunity

CRISPR-Cas systems serve as not only a powerful immune weapon for bacteria to resist foreign invaders, but also an impactful regulator of the expression of endogenous genes, exemplified by the *E. coli* CRISPR-Cas. CRISPR-Cas systems of *E. coli* regulate endogenous genes and signaling pathways, which may be active only under specific conditions through a variety of mechanisms, such as DNA interactions, mRNA sequence targeting, and transcription or elongation regulation [80]. Multiple studies demonstrated bacteria containing self-targeting spacers (STS), for instance, CRISPR spacers targeting protospacers on the same genome to exhibit adverse effects. STSs are thought to provoke autoimmunity, an unwanted side effect of CRISPR-Cas defense, while the newer concept points out perhaps a regulatory mechanism for gene expression. Nobrega et al. found the existence of STS in all CRISPR-Cas types and 20% of all CRISPR-carrying bacteria. In particular, up to 40% of I-B and I-F CRISPR-Cas systems in *E. coli* contained STS.

Intriguingly, although STSs are almost ubiquitous, they hardly cause serious adverse consequences to the bacteria themselves. This may be related to the presence of anti-CRISPR (Acr) proteins, which may serve as a self-regulatory mechanism for the avoidance of autoimmunity [81]. This phenomenon ponders many scientists and encourages further understanding of the underlying regulatory mechanisms critical to maintaining the stable equilibrium of bacteria by countering or fine-tuning the self-targeting elements. Recently, it has been demonstrated that type II-B CRISPR-Cas9 systems and type V-A CRISPR Cas12a systems inhibit transcription and defense through CRISPR RNA (crRNA) guidance and limit complementarity between target genes [82]. The CRISPR systems can target endogenous genes to affect the physiological function of bacteria. CRISPR-Cas systems that target the bacterial genome are thought to have a lethal effect on the host, but a small number of bacteria can escape this CRISPR-Cas attack through genome remodeling. In the model organism *Streptococcus thermophilus* DGCC710, the escape of self-targeting of endogenous type I and type II CRISPR-Cas systems is primarily the result of the removal of low-frequency defective plasmids in the targeted spacer [83]. This helps explain that side effects are generally limited with CRISPR-Cas self-targeting, leading to the reconciliation that self-attack perhaps in some instances may serve as potential self-conservation or protection mechanisms by removing unimportant or decayed genetic materials in the genome.

To study roles of acetyl-CoA in microbial energy and material metabolism, researchers have used a tunable CRISPR interference (CRISPRi) system for multiplex repression of the expression of endogenous genes (*pta*, *frdA*, *ldhA*, and *adhE*) in *Escherichia coli*, leading to reduced end products of carbon metabolism and carbon flux and increased acetyl-CoA. These results demonstrated that the CRISPRi system could serve as a tool for simultaneously regulating bacterial genes to analyze biosynthetic pathways, namely bacterial metabolism. Although this method provides a technical platform for other metabolic research, potentially incomplete silencing CRISPR may partially indicate a possible function attributing to the CRISPR’s natural features, endogenous regulation of metabolisms, which needs to be investigated [84].

### Complex roles of CRISPR-Cas in influencing host immunity

*cas3* of *S. enterica* may play an active role in its virulence, including cell penetration and animal pathogenicity, by impacting several genes involved in QS, flagellum, and SPI-1-T3SS systems [62]. Additionally, Cas9 may also promote the invasion and proliferation of *Neisseria meningitidis* in hosts, suggesting that the role of type II CRISPR-Cas in virulence modulation varies with bacteria and their respective environments [21]. *C. jejuni* seems to contain a complex CRISPR system in the regulation of pathogenicity; and mutants lacking *csn*, a type II CRISPR-Cas marker, increase swarming and reduce virulence during infection of the host. The lack of Csn also promoted the binding of antibodies to cell surface molecules, indicating that Csn is involved in regulating the conformation and/or composition of the cell membrane [70]. In addition, CRISPR-Cas systems in *Shigella* are widespread, and *Shigella* strains without CRISPR1 (a specific CRISPR array in *Shigella* genome) exhibit higher pathogenicity and virulence [85].

**Fig. 4:**
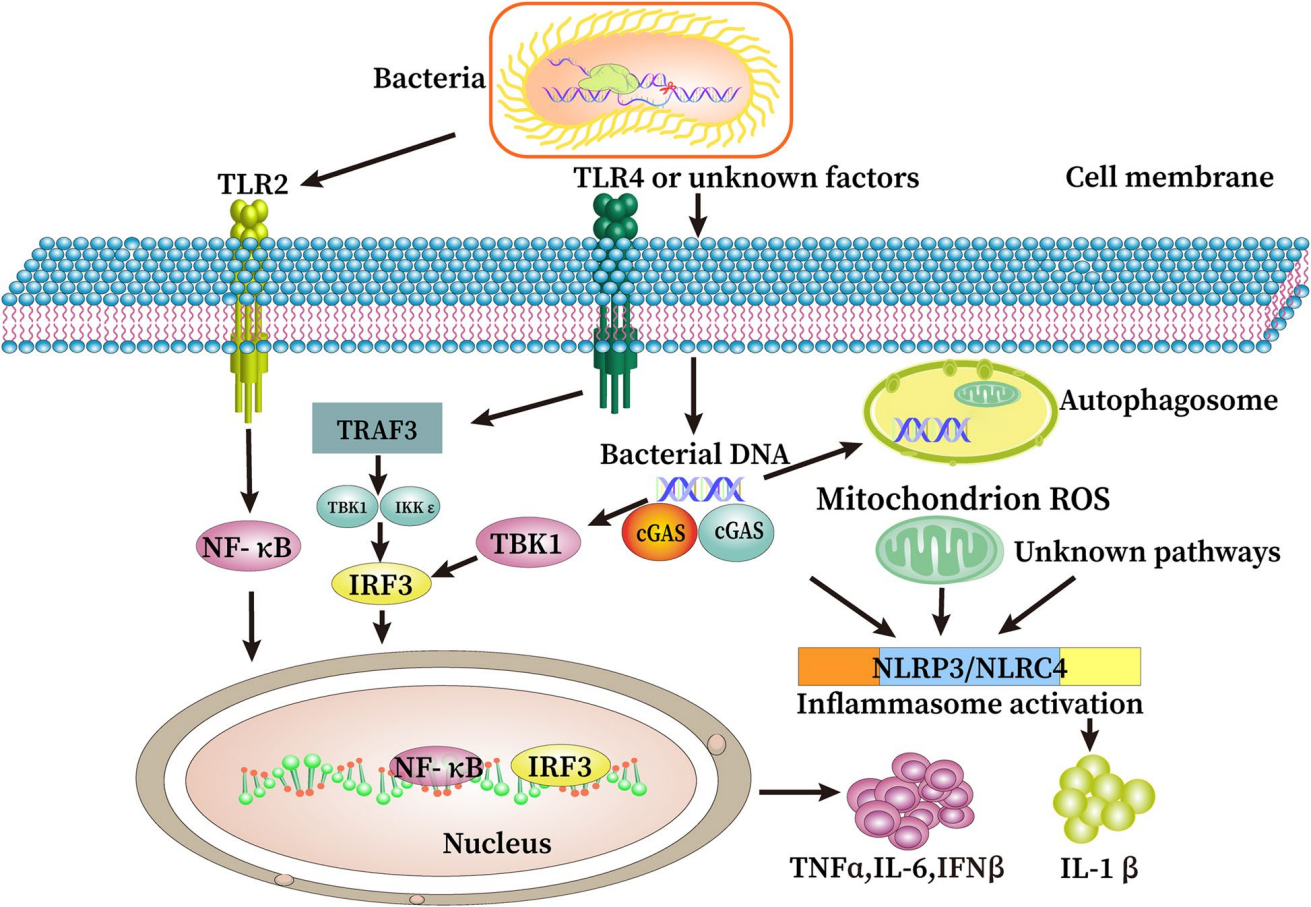
CRISPR-Cas impacts host defense by regulating several signals involving innate immunity and inflammatory responses. CRISPR-Cas may participate in the recognition of pattern recognition receptors (PRRs) (e.g., TLR2, TLR4 or other unknown PRRs) and the activation of their downstream signaling pathways. The downstream effects include altered levels of mitochondrion ROS, inflammasome, autophagy, depletion of CRISPR-Cas in *P. aeruginosa* strain PA14 leads to exaggerated inflammation and organ damage through rapid nuclear translocation of the transcription factor NF-κB after PRRs recognition

On the contrary, CRISPR-Cas systems may contribute to increased invasive potency and knockdown of them may lead to lower virulence. In a *Salmonella pullorum* strain, CRISPR components contributed to the bacterial dispersion and survival in hostile habitats, manifesting increased virulence [86]. Likewise, *Legionella pneumophila* requires CRISPR-Cas for pathogenesis; for instance, the *cas2* gene seemed to be responsible for causing infection in amoebae. However, mutant strains lacking *cas9, cas1,* and *cas4* did not show apparent roles in the infection of macrophages and aquatic amoeba indicating that each of Cas proteins plays a distinct role in pathogenesis [71]. Hence, the detailed mechanisms of Cas2 in regulating bacterial virulence remain unclear and warrant further research. A study showed that RNase activity of Cas2 may be related to the virulence mRNA expression by activating RNase [72]. Wu et al. found that *P. aeruginosa* type I CRISPR-Cas system affects bacterial virulence, the absence of type I CRISPR-Cas system led to a more serious disease phenotype in the host. Compared with WT *P. aeruginosa* (PA14), the expression of NLRC4 inflammasome and the level of pro-inflammatory cytokine IL-1β in lung of infected mice significantly increased in total CRISPR-Cas regions (TCR) knockout bacteria group. Concurrently, it was also discovered that type I CRISPR-Cas system involved in the reactive oxygen species of mitochondria and participates in the activation of inflammasome by influencing the release of mtDNA [68] (Fig. 4).

Together, these studies suggest that different CRISPR-Cas systems play distinct roles in bacterial pathogenesis upon infecting hosts, which requires continued investigations. Another possibility is that Type I-E CRISPR-Cas systems may only partially modulate the transcription of certain endogenous mRNAs as a means to gauging and modulating network interactions within the bacteria (e.g., *E. coli*) to combat phages through naïve adaptation. This type of CRISPR self-regulation could cause a bystander effect that may also indirectly change the landscape of host immune response, which may not be true endogenous targeting and impact interpretation of how the host responds to bacterial infection [80]. Overall, although a large number of literature pointed out that the potential endogenous targeting is linked to immune reactions in the host, further experimentation is urgently required to address the complexity and underlying mechanisms with various *cas* genes in regulating virulence in the broader bacterium kingdom. Collectively, these findings suggest that different CRISPR-Cas systems play distinct roles in bacterial pathogenesis upon infecting hosts, which requires continued and deeper investigations.

### Reconciliation with nucleic cleavage in endogenous targeting

The widespread existence of CRISPR-Cas systems in prokaryotes suggests that the possibility of self-targeting to the homologous regions in their genomes may cause potential autoimmunity or modulate defense mechanisms [19, 87]. Sequencing analyses unveil that the homologous regions are not generally long enough to cause extensive self-destruction and unlikely induce organism death. Further, other intrinsic mechanisms, such as homologous recombination, may provide the plasticity and remodeling of bacterial genomes, and thereby leading to evasion of internal genome targeting [83].

Although the evidence for regulating endogenous gene expression by CRISPR-Cas systems to alter bacterial behaviors instead self-killing is mounting, how this occurs is largely unclear. Particularly, the evidence for direct nuclease cleavage of bacterial endogenous genes on genomic loci or transcripts is scarce, and often difficult to gain solid verifications in vivo due to the current technical hindrances.

The most extensively-studied Cas9 was earlier demonstrated to only cleave DNA but is now implicated in cleavage of both DNA and RNA depending on bacterial species and strains as well as the experimental conditions [88]. For example, the *C. jejuni* Cas9 (CjCas9) is implicated in binding and cleaving complementary endogenous mRNAs in a crRNA-dependent manner by Dugar et al., who recently reported that about hundreds of transcripts were co-immunoprecipitated with CjCas9 through their base-pairing with crRNAs, and, importantly, some of these RNAs could be enzymatically cleaved at the predicted binding sites [79]. Mutational analyses show that the cleavage was crRNA and tracrRNA dependent and requiring the CjCas9 HNH domain.

The study conducted in Type II CRISPR-Cas of *F. novicida* initially reported cleavage of mRNA of BLP, but later the authors found that it was hard to reproduce the data [18, 21]. The other study performed in our laboratory found the Cas3-mediated cleavage of *lasR* mRNA in *P. aeruginosa* strain PA14 by Type I-F CRISPR-Cas systems that were thought to only target and cleave DNA (Fig. 3). However, this cleavage seemed to be irreproducible through the purified protein system by the third party [89], which might be due to the impure protein mixture and the experiment conditions and reagents that were utilized in our initial report [24].

Although Cas9 is shown to cleave endogenous RNA, Wiedenheft and colleagues have indicated the irreproducibility of RNA cleavage by Type I-F CRISPR systems in our early report [79, 89]. We extremely appreciate the challenge to our in vitro RNA cleavage assay, and would like to sincerely apologize to the scientific community for the inconvenience this observation may have caused. Furthermore, the RNA cleavage has never been tested in cells or animal models, which require additional validation for possibilities targeting this RNA transcript or other endogenous RNA (potentially DNA) sequences. Nevertheless, we were able to reproduce the increased expression of *lasR* in the PA14 strain with deletion of Total CRISPR-Cas regions (TCR) by independent lab members [24, 68]. In addition, the deletion of *cas3* in another lab could not detect strong inhibition of *lasR* mRNA [90], which might be due to several differences, such as the strains used, experimental settings, as well as mutation region (total *CRISPR* vs. *cas3*) and approaches used for generating these mutations.

Nevertheless, nuclear acids cleavage assays thus far were only shown in experimentally reconstituted in silico, there was no direct evidence demonstrating that endogenous targeting occurs in vivo in bacteria, in mammalian cells, or whole animals after the bacterial invasion. Therefore, the studies regarding CRISPR-mediated endogenous gene regulation in bacteria or pathogenic effects on mammalian hosts might not be associated with nucleic acid cleavage but are indirectly related to the transcription, expression, or decay of the underlying gene and nearby genes. In addition, endogenous targeting might be attributed to other unrecognized mechanisms. The function and regulation of CRISPR-Cas systems are highly diverse, surprising, and somewhat unpredictable. It is imperative to further dissect the critical mechanisms of how endogenous genes can be targeted, regulated, and/or cleaved to influence bacterial physiology, virulence, and ultimately impact the courses and potencies of mammalian immune responses, such as phagocytosis and inflammatory responses by professional and unprofessional immune cells.

Previous reports on non-canonical roles in pathogenesis were primarily involved in Type I and Type II CRISPR, a recent study revealed that the Type IIIA system in *M. tuberculosis* (Type III-A system) might be a new player to directly target host responses through secretion of CRISPR-Cas effectors into extracellular compartments. The authors showed that secreted Csm1, 3, 5 and 6, and Cas6 proteins activated immune responses in both cell culture and animal experiments as well as IFN-γ release in T cells and macrophages from active tuberculosis patients [20]. This study and some other observations indicate that pathogens may utilize CRISPR-Cas proteins to exert non-canonical function as virulence factors to retour host immune responses and inflammatory responses to cause more severe host tissue damage [20, 91], which seems irrelevant to endogenous targeting and should be taken into consideration when investigating endogenous CRISPR-Cas targeting for the involvement of host immunity.

## CRISPR-Cas systems influence antibiotic resistance

### CRISPR-Cas and antibiotic resistance

As the immune apparatus found in prokaryotes, CRISPR-Cas systems represent a powerful weapon to counteract against invasive exogenous genetic elements and phages. Remarkably, the structure and function of CRISPR-Cas are also potentially linked to antibiotic resistance. *Acinetobacter baumannii* has strong virulence and increasingly serious drug resistance [92]. Recently, Tyumentseva M et al. studied core features of antibiotic resistance genes through sequence analyses of CRISPR arrays in 12 clinical isolates of *A. baumannii* by using multilocus sequence typing schemes. Their results showed that clinical isolates of strong drug resistance genes contain less CRISPR arrays and active *cas* genes, suggesting that CRISPR-Cas systems can inhibit the drug resistance in *A. baumannii* [93].

*Cas9* mutations in *C. jejuni* rendered strong sensitivity to antibiotics compared to WT strains, suggesting that Cas9 likely promotes antibiotic resistance [94]. A recent clinical study with 168 carbapenem-resistant *Klebsiella pneumoniae* (CRKP) strains from inpatients identified both CRISPR-Cas-positive and CRISPR-negative isolates. The results showed that patients infected by strains harboring the type I-E^*^ CRISPR-Cas system manifested increased mortality vs. patients infected by CRISPR-negative isolates. In the CRISPR-negative strains, the frequency of carbapenemase gene *KPC-2*, an important drug resistance gene in *K. pneumoniae*, was the highest compared to CRISPR-containing strains. This study suggested that there is a reverse correlation between CRISPR systems and antibiotic-resistance, providing strong impact on antibiotic resistance in the clinical strains [64].

The possible reason for CRKP strains with positive CRISPR-Cas system still obtain carbapenem gene is that different CRISPR-Cas systems have different spacers, CRISPR-Cas systems interferes with the acquisition of carbapenem gene, which not only depends on the spacer matching in CRISPR-Cas system, but also is affected by the PAM recognition of foreign carbapenem gene. The abuse of carbapenem antibiotics will help bacteria obtain carbapenemase gene [95]. Some CRISPR-Cas positive strains have been inactivated for a long time because of the existence of self-targeting spacers [96]. There are anti-CRISPR proteins (Acrs) in bacteria to inhibit the immune function of CRISPR-Cas systems [97]. This may be associated with one of the common antibiotic resistance mechanisms by hiding bacteria within biofilms (up to 1000 times compared to their planktonic counterparts), often resulting in persistent infection and very difficult to eradicate [98].

From the clinical prospect, the ability to promote antibiotic resistance by CRISPR-Cas is considered as a disadvantage by upregulating biofilm and thereby leading to resistance to phage therapy. It should be noted that phage therapy is becoming an important alternative or auxiliary therapy for multidrug-resistant bacterial infections. A thorough analysis of the relationship between CRISPR-Cas and bacterial antibiotic resistance would improve understanding of drug-resistance mechanisms and potentially reveal new strategies for the prevention and treatment of refractory infection [23]. Recently discovered Acrs can counteract the CRISPR-Cas immunity towards phages and thus hold promise for clinically combatting bacterial infection [99–101].

### CRISPR-Cas as targets to counteract antibiotic resistance

On the other hand, CRISPR-Cas systems may be exploited to reduce drug resistance and augment the mammalian defense against infection. For instance, targeting CRISPR-Cas DNA sequences interferes with the horizontal transfer of a specific *Staphylococcal* resistance gene, limiting the spread of drug-resistant pathogens. This may represent a potential drug therapy target and further understanding detailed mechanism is a must for biotechnological development [102, 103]. Interestingly, some bacterial strains lacking CRISPR-Cas are inclined to acquire antibiotics resistance genes, and lack of CRISPR-Cas appears to render bacteria susceptible to bacteriophage attack. This phenomenon is further compounded with variable observations in different types of bacteria (with varying CRISPR systems) and/or environmental settings [60, 90]. The study of phylogenetic distribution in *P. aeruginosa* showed that CRISPR-Cas systems acquired drug-resistant genes through reconstructing auxiliary genome distribution [104]. The researchers analyzed 75 isolates of *Salmonella* from chicken farms, found that nearly 80% of the strains were resistant to one or more antibiotics. Through counting the Spearman’s rank correlation of the CRISPR-Cas system and antibiotic resistance genes of the strains, the study unveiled that the CRISPR loci were closely related to the antibiotic resistance genes [105].

### CRISPR-Cas systems block genetic transfer

Horizontal gene transfer is the main mode of causing bacterial drug resistance, leading to the spread of superbug infection [106, 107]. A study uncovered that multidrug-resistant enterococci lacked CRISPR-Cas elements and showed that the increased resistance of *E. faecalis* to antibiotic was due to the loss of CRISPR-Cas locus in the gene sequence. At the same time, it was also found that *E. faecalis* had only CRISPR locus without Cas protein and interestingly, its genome still contains rich antibiotic resistance genes, suggesting that only a certain component of CRISPR-Cas may be sufficient to alter bacterial drug resistance transmission [108].

Another recent study with *K. pneumoniae* revealed that the antimicrobial resistance of CRISPR-Cas positive strains was lower than those negative strains [109]. Importantly, type I-E systems might alter the spread of bla_KPC_ in *K. pneumoniae* [110]. CRISPR-Cas systems may inhibit the acquisition of *bla*_KPC_ plasmids contributing to carbapenem resistance in *K. pneumoniae* strains because of possessing CRISPR spacers that are in high similarity to known multidrug-resistance plasmids. Correspondingly, resistance to *bla*_KPC_ plasmid in the *K. pneumoniae* strain hosting CRISPR-Cas may be abolished through removal of the CRISPR-Cas cassette [109]. These studies further confirm the complexity of CRISPR-Cas function in drug resistance, which warrants further investigation.

### Mechanisms of CRISPR-Cas-assisted drug resistance

Interestingly, most non-pathogenic *S. epidermidis* strains lack CRISPR sequences, while a clinical isolate, RP62a containing CRISPR sequences, showed resistance to the invasion of exogenous genes to a certain extent [111]. To examine the mechanistic feature of CRISPR in drug resistance, the beta-lactam plasmid pG0400 in *S. aureus* is deactivated and transformed into CRISPR *S. epidermidis* strains or control strains. The results showed that CRISPR-Cas interference significantly impaired horizontal transfer of drug-resistant plasmids (from *S. aureus* to *S. epidermidis*), which may represent a mechanism of blocking drug resistance [102]. Some CRISPR spacers of *Mollicutes* could target bacterial or phage genes, which may serve as part of integrative and conjugative elements (ICE) [112].

Recently, *E. faecalis* as a model organism and a natural colonizer of the mammalian intestine that contains pheromone-responsive plasmids (PRPs) could mediate inter- and intra-species transfer of antibiotic resistance genes. WT bacteria containing CRISPR-Cas can block the transfer and dissemination of antibiotic-resistant plasmids but the Δ*cas9* mutant strain cannot [113]. Overall, we only know the tip of an iceberg about the role of CRISPR-Cas in regulating drug resistance. Critically, we point out that multiple approaches, especially novel ones, are highly desired to further unravel the functions for CRISPR-Cas systems in antibiotic resistance in depth.

## Conclusions and perspectives

In summary, the role of CRISPR-Cas systems has gone beyond the canonical function, adaptive immunity. One of these non-canonical functions of the CRISPR-Cas systems in regulating bacterial genes that are involved in invading mammalian hosts is described as an example in Figs. 3 and 4. In addition to adaptive immunity, the function of CRISPR-Cas systems is gradually unfolded by modifying the expression of some critical bacterial genes, thereby impacting their physiological characteristics. Numerous studies have emphatically demonstrated that bacterial functions may be affected by CRISPR-Cas systems. Understanding CRISPR-Cas-mediated gene regulation is mainly derived from unbiased screening for regulators of distinct phenotypes, such as virulence or bacterial physiology behaviors [114]. Importantly, the knowledge of CRISPR-Cas systems is constantly evolving at an enormous pace. Recently, type VI CRISPR-Cas systems and their related protein Csx27 have been implicated in similar regulation of virulence in *E. coli*. Csx27 forms a membrane channel of solid DNA and may be involved in the ubiquitin signaling pathway, which serves as new machinery for bacterial degradation of exogenous DNA [115]. Therefore, it might be inferred that the ever-expanding CRISPR-Cas systems regulate far more genes than have been identified to date while through a spectrum of different mechanisms.

Dissecting these non-canonical functions of CRISPR-Cas systems has broadened the understanding of bacterial physiological functions and regulatory networks. Furthermore, creative strategies for studying interaction and inter-regulation between CRISPR-Cas systems and bacterial virulence genes can also guide researchers to design experiments for fundamental research or biotechnological application, such as CRISPR-targeting antimicrobial agents. It is worth mentioning that harnessing the native type I-F CRISPR-Cas system has been implemented to delete the *AMR* (antimicrobial resistance) gene in multidrug-resistant *P. aeruginosa* [116]. Improved understanding of the influence of CRISPR-Cas systems on bacterial physiology and pathogenicity will have a profound impact on the control of bacterial infection as well as continuously providing new biotechnological tools to control genetic diseases, cancers and so on.

## Data Availability

Not applicable.

## Abbreviations

CRISPR-Cas: Clustered regularly interspaced short palindromic repeats-CRISPR associated proteins
EPS: Extracellular polymeric substances
PAM: Protospacer-adjacent motif
sRNAs: Small non-coding RNAs
c-di-GMP: Cyclic diguanylate monophosphate
AI: Autoinducers
AHL: Acyl homoserine lactone
QS: Quorum sensing
ICU: Intensive care unit
BLP: Bacterial lipoprotein
PAMP: Pathogen-associated molecular pattern
STS: Self-targeting spacers
CRKP: Carbapenem-resistant *Klebsiella pneumoniae*
Acrs: Anti-CRISPR proteins
ICE: Integrative and conjugative elements
PRPs: Pheromone-responsive plasmids
WT: Wild type
